# Performance of ambiguity-resolved detector for GNSS mixed-integer model

**DOI:** 10.1007/s10291-024-01806-4

**Published:** 2025-03-08

**Authors:** Chengyu Yin, P. J. G. Teunissen, C. C. J. M. Tiberius

**Affiliations:** 1 Department of Geoscience and Remote Sensing, Delft University of Technology, Delft, The Netherlands; 2 Department of Infrastructure Engineering, The University of Melbourne, Melbourne, Australia; 3 GNSS Research Centre, Curtin University, Perth, Australia

**Keywords:** GNSS, Mixed-integer model, Ambiguity resolution, Model validation, Monte Carlo simulation

## Abstract

Teunissen (J Geod 98(83):1–16, 2024) proposed the ambiguity-resolved (AR) detection theory for GNSS mixed-integer model validation. In this contribution, we study the performance of the AR detector through analysis and simulation experiments and compare it with the ambiguity-float (AF) and ambiguity-known (AK) detectors. We describe how the detectors can be implemented and how to evaluate their performance by computing the power as functions of the model misspecifications’ size. We present two simulation experiments with single- and dual-frequency GPS models and demonstrate that the AR detector can provide a larger detection power than the AF detector, even if the success rate is not close to one. Then, we obtain power functions over 25 user locations with five observation models and 72 satellite geometries per location per model. We find that the AR detector increases the detection probability of ionosphere and troposphere delays by 47% and 60% on average when the success rate is larger than 97.5% and the level of significance is 0.01. We also find the AR detection power to be larger than that of the AF detector in case of multi-dimensional misspecifications.

## Introduction

The Global Navigation Satellite System (GNSS) observation models connect the observables and the unknown model parameters. Unmodeled effects may misspecify the assumed model, which can seriously deteriorate the estimation results when they remain unnoticed (Misra and Enge 2011; Hofmann-Wellenhof et al. 2012; Teunissen and Montenbruck 2017). Therefore, the validation of the observation model is essential in GNSS data processing. The detection, identification, and adaptation (DIA) procedure has been widely used in model validation (Baarda 1968; Kok 1984; Teunissen 2000). Detection is the first step in this procedure, where an overall model test is performed to diagnose if a model misspecification occurs.

Currently, the detection of GNSS mixed-integer model misspecifications is either based on the ambiguity-float (AF) detector or the ambiguity-known (AK) detector (Teunissen 2024). The AF detector is applied when the ambiguity is not resolved (fixed) to an integer vector. The ambiguity is considered to be an *unknown real* vector, and knowledge of its integer property is then not used to benefit detection. The AK detector, on the other hand, can be applied when the ambiguity is completely *known*. In practice, ambiguity resolution is carried out when the success rate is very close to one (e.g., > 0.999), and the resolved ambiguity is commonly assumed to be known and consequently treated as a deterministic quantity (Feng et al. 2009; Wang et al. 2022; Zhang et al. 2023), although it is a random estimator. Teunissen (2024) proposes the ambiguity-resolved (AR) detection theory, in which the ambiguity is treated as an *unknown integer* vector. By considering the distributional property of the resolved ambiguity, the AR-detector enables ambiguity resolution to contribute to model validation even if the success rate is not close to one. The distribution of the AR test statistic cannot be written in a closed form due to the discrete nature of the resolved ambiguity. Numerical simulations and analysis are necessary to understand the performance of the AR detector for detecting GNSS model misspecifications.

We evaluate and compare the performance of the AF, AK, and AR detectors for the single-differenced (SD) common-oscillator model and the short baseline double-differenced (DD) model. The SD model with a common oscillator is used in attitude determination, which can be affected by constant biases (Keong 1999; Chen 2016). The short baseline DD model is one of the commonly used relative positioning models, in which the pseudorange and carrier phase observables from more than one receiver are combined to eliminate or reduce the common errors between the receivers (Leick et al. 2015; Odijk 2017). The relative positioning models can be formulated with undifferenced (UD), SD, or DD observables collected from a single baseline or a network (Tiberius 1998; Odijk 2017). We use the short baseline DD model as an example to conduct experiments and compare the performance of the detectors. Similar performance can be expected for the models formulated with UD and SD observables, as well as the atmosphere-corrected network-RTK and PPP-RTK user models, which are intrinsically equivalent to the short-baseline DD model given that the user observation setup is the same and the variance-covariance (vc) matrix of the corrections are appropriately considered (Teunissen and Khodabandeh 2015; Odijk et al. 2016; Kouba et al. 2017). We focus on the validation of relative positioning models, which are vulnerable to blunders in pseudorange observables, carrier phase outliers due to multipath or faulty corrections and cycle slips (Braasch 2017; Duan et al. 2024), and ionosphere and troposphere delays that are not fully canceled or corrected (Ahn et al. 2006; Lawrence et al. 2006; Huang and van Graas 2007; Wanninger 2004; Hernández-Pajares et al. 2011; Dao et al. 2024). These misspecifications are modeled by means of the alternative hypotheses $\:{H}_{a}$, for which the detection power will be analyzed.

This contribution starts with the theoretical background: the observation models under the null and alternative hypotheses are described, and the ambiguity resolution and misspecification detection theory for the GNSS mixed-integer models is reviewed. After that, we describe how to carry out the mixed-integer model detection and how to obtain the statistical power of the detectors. The critical value and power of the AR detector can only be obtained by the Monte Carlo method; thus, we introduce how to evaluate the uncertainty of the Monte Carlo simulations. It is followed by the experiment section that compares the power of detectors for one- and multi-dimensional model misspecifications, where the superiority of the AR detector to the AF detector is shown, and the irregular shapes of the AR power functions are explained. Finally, the summary and conclusions are presented.

## Review of theory

### Differential observation model

Assume $\:s+1$ satellites from the same constellation are tracked by the rover and reference receivers on $\:f$ frequencies for $\:k$ epochs. The linearized double-differenced (DD) GNSS observation model can be written as

1 $$\begin{aligned}&{H}_{0}:\:\:\:\:E\left\{\underline{y}\right\}=Aa+Bb,\:D\left\{\underline{y}\right\}={Q}_{yy},\\&{H}_{a}:\:\:\:\:E\left\{\underline{y}\right\}=Aa+Bb+Cc,\:D\left\{\underline{y}\right\}={Q}_{yy},\end{aligned}$$

where $\:{H}_{0}$ and $\:{H}_{a}$ denote the null and alternative hypotheses, respectively. $\:E\{\cdot\}$ is the expectation operator and $\:D\{\cdot\}$ is the dispersion operator. The underline $\:'\underline{\cdot}'$ denotes a random variable or vector. $\:\underline{y}={[{\underline{\varphi}}^{T},{\underline{p}}^{T}]}^{T}\in{\mathbb{R}}^{2sfk}$ contains the double-differenced carrier phase $\:\underline{\varphi}$ and pseudorange $\:\underline{p}$ and we assume them to be normally distributed. $\:{\mathbb{R}}^{\left(*\right)}$ represents a $\:\left(*\right)$ dimensional real space. With this definition of $\:{H}_{a}$, we consider only misspecifications on the mean of $\:\underline{y},$ which are modeled by design matrix $\:C$ and vector $\:c\in{\mathbb{R}}^{q},$ with $\:q$ the dimension of the misspecification under $\:{H}_{a}$. $\:a\in{\mathbb{Z}}^{sf}$ is the unknown integer ambiguity vector and $\:b\in{\mathbb{R}}^{3}$ is the real-valued baseline vector.$\:\:{\mathbb{Z}}^{\left(*\right)}$ represents a $\:\left(*\right)$ dimensional integer space. The design matrix $\:\left[A,B,C\right]$ is assumed to be of full column rank.

The design matrix for the ambiguity vector is

2 $$\:A={\left[\begin{array}{cc}1&0\end{array}\right]}^{T}\otimes \mathbf{1}_{k}\otimes\text{d}\text{i}\text{a}\text{g}\left({\lambda}_{1},\:\cdots,\:{\lambda}_{f}\right)\otimes{I}_{s},$$

where ⊗ denotes the Kronecker product, $\:\mathbf{1}_{k}\:$is a $\:k\times1$ vector with values of $\:1,$ ‘$\:\text{d}\text{i}\text{a}\text{g}$’ refers to a diagonal matrix, $\:{\lambda}_{f}$ is the wavelength of the $\:f$-th frequency, and $\:{I}_{s}$ is an $\:s\:\times\:\:s$ identity matrix.

The design matrix for the baseline is

3 $$\:B=\mathbf{1}_{2}\otimes{M}_{k}\otimes\mathbf{1}_{f}\otimes\left({D}^{T}G\right),$$

with $\:{M}_{k}=\mathbf{1}_{k}$ for a stationary receiver and $\:{M}_{k}={I}_{k}$ for a moving receiver; $\:{D}^{T}=\left[-\mathbf{1}_{s},{I}_{s}\right]$ the $\:s\:\times\:\:(s\:+\:1)$ between-satellite differencing matrix ($\:{D}^{T}={I}_{s+1}$ for the SD model in which the between-satellite differencing is not conducted); $\:G$ the $\:(s\:+\:1)\:\times\:\:3$ geometry matrix contains the unit vectors from satellites to receivers. In this contribution, we consider the instantaneous and short time span cases where $\:G$ is practically time-invariant.

The variance-covariance (vc) matrix of observable $\:\underline{y}$ is

4 $$\:{Q}_{yy}=\text{d}\text{i}\text{a}\text{g}\left({\sigma}_{\varphi}^{2},{\sigma}_{p}^{2}\right)\otimes{R}_{k}\otimes{I}_{f}\otimes2{D}^{T}{W}^{-1}D,$$

$\:{\sigma}_{\varphi}^{2}$ and $\:{\sigma}_{p}^{2}$ are the zenith-referenced undifferenced phase and pseudorange variances; we assume that the phase and pseudorange observables are uncorrelated. The non-diagonal elements of the $\:k\times k$ matrix $\:{R}_{k}$ account for the time correlation between the observables from different epochs, and its diagonal values are 1 (Odijk and Teunissen 2008). With the $\:f\times f$ identity matrix $\:{I}_{f},$ we assume the observables from different frequencies are uncorrelated and of the same precision. $\:W=\text{d}\text{i}\text{a}\text{g}\left({w}_{1},\:\:\cdots,\:\:{w}_{i},\:\:\cdots\:,\:\:{w}_{s+1}\right)$ contains the elevation-dependent weights for each satellite (Euler and Goad 1991),

5 $$\:{w}_{i}=1/{\left({a}_{0}+{a}_{1}\text{e}\text{x}\text{p}\left(-{E}^{i}/{E}_{0}\right)\right)}^{2},$$

where $\:{E}^{i}$ is the elevation of the $\:i$-th satellite in degrees. Examples of the model coefficients $\:{a}_{0},\:\:{a}_{1}$ and $\:{E}_{0}\:$can be found in (Euler and Goad 1991; Jin and de Jong 1996). $\:\:{a}_{0}=1,\:{a}_{1}=10$ and $\:{E}_{0}=10$ degrees are used in our simulation experiments.

When the DD model contains observables from two constellations, the design matrices and vc-matrix can be stacked as follows,

6 $$\:{A}^{12}=\left[\begin{array}{cc}{A}^{1}& \\ &{A}^{2}\end{array}\right],\:\:{B}^{12}=\left[\begin{array}{c}{B}^{1}\\{B}^{2}\end{array}\right],\:\:{Q}_{yy}^{12}=\left[\begin{array}{cc}{Q}_{yy}^{1}&\\&{Q}_{yy}^{2}\end{array}\right],$$

where $\:{A}^{i},\:{B}^{i}$ and $\:{Q}_{yy}^{i}$ are the design matrices and vc-matrix for the *i*-th constellation. The observables are differenced within each constellation, and the receiver hardware delays are canceled; thus, inter-system biases do not appear in the model.

To evaluate the performance of the detectors for a specific model misspecification, we consider the observation model under $\:{H}_{a},$ in which $\:C$ models a misspecification of size $\:c$. We evaluate the detection performance of four misspecifications with the DD model and of one misspecification with the SD common oscillator model. The design matrices for the one-dimensional misspecification are given below, and they can be concatenated when we work with multi-dimensional misspecifications, e.g., blunders in several pseudorange observables.

Although most errors and delays are eliminated or corrected in relative positioning models, pseudorange multipath delays may appear and affect the estimation accuracy, ranging from several meters to even 100 m in most severe conditions (Braasch 2017). The design matrix for a blunder in pseudorange observable of the *i*-th satellite, *j*-th frequency, *k*-th epoch is

7 $$\:C=\left[\begin{array}{c}0\\1\end{array}\right]\otimes\:{c}^{k}\otimes\:{c}^{j}\otimes\:{D}^{T}{c}^{i},$$

where $\:{c}^{i}$ is a canonical vector with its $\:i$*-*th entry equal to one and zero for others.

Phase observables may contain outliers and cycle slips. Additionally, phase bias corrections in the network-based scenario may contain errors that can be interpreted as phase outliers in the user model at the centimeters level (e.g., Duan et al. 2024). To evaluate the performance of the detectors for phase outliers, we use the model with two epochs, and we assume the outlier or cycle slip appears in the observables of the second epoch. For the phase outlier from the *i*-th satellite, *j*-th frequency, the corresponding design matrix is written as

8 $$\:C\:=\left[\begin{array}{c}1\\0\end{array}\right]\otimes\left[\begin{array}{c}0\\1\end{array}\right]\otimes{c}^{j}\otimes{D}^{T}{c}^{i}.$$

The troposphere delay can be separated into dry and wet constituents, of which the dry component can be modeled with high precision. In relative positioning models, the wet delay could be canceled or corrected in most cases. However, during severe weather conditions, troposphere anomalies can cause delays ranging from several centimeters to several decimeters in the differenced/corrected observables (Ahn et al. 2006; Lawrence et al. 2006; Huang and Van Graas 2007). The design matrix for the troposphere delay at the *k*-th epoch is

9 $$\:C=\left[\begin{array}{c}1\\1\end{array}\right]\otimes{c}^{k}\otimes\mathbf{1}_{f}\otimes\:{D}^{T}{\left[\begin{array}{ccc}m\left({E}^{1}\right)&\:\cdots&\:m\left({E}^{s+1}\right)\end{array}\right]}^{T},$$

with $\:m(\cdot)$ the troposphere mapping function. $\:m\left(E\right)=1/\sin\left(E\right)$ is used in our simulations (Hobinger and Jakowski 2017). The misspecification $\:c$ in this model is the differential zenith wet delay in meters.

Similar to the troposphere delay, the differential ionosphere delay can be ignored under the nominal condition, while this is not the case under anomalous conditions, for example, in the presence of ionosphere disturbance (Wanninger 2004; Hernández-Pajares et al. 2011) or when the ionosphere spatial gradients are hundreds of millimeters per kilometer, which can lead to differential ionosphere delays at the decimeter level (Pullen et al. 2009). The performance of the AR detector for ionosphere delays of satellite $\:i$ on epoch $\:k$ is evaluated with the design matrix

10 $$\:C=\left[\begin{array}{c}-1\\\:1\end{array}\right]\otimes\:{c}^{k}\otimes\:\mu\:\otimes\:{D}^{T}{c}^{i},$$

where $$\:\mu={\left[\begin{array}{ccc}\frac{{\lambda}_{1}^{2}}{{\lambda}_{1}^{2}}&\cdots&\frac{{\lambda}_{f}^{2}}{{\lambda}_{1}^{2}}\end{array}\right]}^{T}$$. The misspecification $\:c$ in this model is the size of the differential ionosphere delay of the first frequency in meters. The value can be converted to TECU by multiplying the delay (in meters) by $\:\frac{2{f}_{1}^{2}}{80.6\times\:{10}^{16}}\approx\:6.1587\:\text{T}\text{E}\text{C}\text{U}/\text{m}$.

The between-receiver single differenced model with a common oscillator is used for attitude determination (Chen 2016). This model can be affected by constant biases, including the initial bias and the line bias caused by the different lengths and materials of the cables. Constant biases are at the centimeter level and are common for all signals (Keong 1999); thus, all entries of the design matrix are one,

11 $$\:C=\left[\begin{array}{c}1\\1\end{array}\right]\otimes\mathbf{1}_{k}\otimes\mathbf{1}_{f}\otimes\mathbf{1}_{s+1}.$$

The SD functional and stochastic models have the same structures as the DD model, where the between-satellite difference matrix $\:{D}^{T}$ is replaced by an identity matrix.

## Ambiguity resolution

Ambiguity resolution is the key step in GNSS model validation with the AR detector (Teunissen 2024), which is a mapping from a float ambiguity estimator to a resolved ambiguity estimator, denoted as $\:\check{\underline{a}}=\mathcal{I}\left(\hat{\underline{a}}\right)$. Three classes of integer ambiguity estimators have been developed (Teunissen 2017): integer estimator, integer aperture estimator, and integer equivariant estimator. In this contribution, we restrict ourselves to the class of integer estimators (Teunissen 1999a), where the ambiguity is always resolved to an integer vector; thus $\:\check{\underline{a}}\in{\mathbb{Z}}^{n},$ with $\:n$ the dimension of the ambiguity vector.

The resolved integer ambiguity $\:\check{\underline{a}}$ is obtained in two steps. First, the integer property of the ambiguity vector is ignored. The float estimator $\:\hat{\underline{a}}$ and its vc-matrix $\:{Q}_{\hat{a}\hat{a}}$ are

12 $$\:\hat{\underline{a}}={\bar{A}}^{+}\underline{y},\:\:{Q}_{\hat{a}\hat{a}}={\left({\bar{A}}^{T}{Q}_{yy}^{-1}\bar{A}\right)}^{-1},$$

where $\:{\bar{A}}^{+}={\left({\bar{A}}^{T}{Q}_{yy}^{-1}\bar{A}\right)}^{-1}{\bar{A}}^{T}{Q}_{yy}^{-1},$ and $\bar{A}={P}_{B}^{\perp}A$ with ${P}_{B}^{\perp\:}=I-B{\left({B}^{T}{Q}_{yy}^{-1}B\right)}^{-1}{B}^{T}{Q}_{yy}^{-1}$. The float ambiguity is resolved to $\:\check{\underline{a}}$ in the second step with one of the integer estimators (Teunissen 1999a), in which the integer least-squares (ILS) estimator has the largest success rate of fixing the float ambiguity to the correct integer (Teunissen 1999b) and thus provides performance closest to the AK detector. The ILS solution can be obtained efficiently by the LAMBDA method (Teunissen 1995), which conducts the integer search based on the decorrelation-transformed ambiguity vector $\:\hat{\underline{z}}$ and its vc-matrix $\:{Q}_{\hat{z}\hat{z}},$

13 $$\:\hat{\underline{z}}\:={Z}^{T}\hat{\underline{a}},\:\:{Q}_{\hat{z}\hat{z}}={Z}^{T}{Q}_{\hat{a}\hat{a}}Z,$$

where $\:{Z}^{T}$ is an admissible ambiguity decorrelation transformation matrix (ibid.).

The success rate of the ILS estimator cannot be computed analytically, and the integer bootstrapping (IB) success rate for the decorrelated ambiguity $\:\hat{\underline{z}}$ provides a tight and easy-to-compute lower bound for it (Teunissen 1998; Verhagen 2003), which is computed as

14 $$\:P({\check{\underline{z}}}_{IB}={Z}^{T}a)=\prod_{i=1}^{n}\left[2{\Phi}\left(\frac{1}{2{\sigma}_{{\hat{z}}_{i|1,\cdots,i-1}}}\right)-1\right],$$

where $\:a$ is the true ambiguity; $\:{\Phi\:}\left(x\right)={\int\:}_{-\infty\:}^{x}\frac{1}{\sqrt{2\pi\:}}\exp\left\{-\frac{1}{2}{v}^{2}\right\}dv$ is the cumulative distribution function (CDF) of the standard normal distribution; the conditional standard deviations $\:{\sigma\:}_{{\hat{z}}_{i|1,\cdots,i-1}}$ are the square root of diagonal values of the diagonal matrix provided by the triangular matrix factorization of $\:{Q}_{\hat{a}\,\hat{a}}$.

## Detection theory

Two detectors that are currently used to detect GNSS model misspecifications are the AF and AK detectors. The AF detector ignores the integer property of the ambiguity and is based on the ambiguity-float residual

15 $$\:\hat{\underline{e}}={P}_{\left[A,B\right]}^{\perp}\underline{y}.$$

The AK detector employs the ambiguity-known residual computed with the known vector $\:a$ and can only be used when the ambiguity is fully known,

16 $$\:\hat{\underline{e}}\left(a\right)={P}_{B}^{\perp}(\underline{y}-Aa).$$

By replacing the known *a* in (16) with the resolved integer ambiguity $\:\check{\underline{a}}$, we obtain the ambiguity-resolved residual,

17 $$\:\check{\underline{e}}=\hat{\underline{e}}\left(\check{\underline{a}}\right)={P}_{B}^{\perp\:}(\underline{y}-A\check{\underline{a}}).$$

Test statistics of the AF, AK, and AR detectors are the corresponding squared norms of the residuals. The AF and AK test statistics and their distributions under $\:{H}_{0}$ are written as,

18 $$\:\begin{aligned} &AF:{\Vert\hat{\underline{e}}\Vert}_{{Q}_{yy}}^{2}|{H}_{0}\sim{\chi}^{2}(r,0),\\ &AK:{\Vert\hat{\underline{e}}\left(a\right)\Vert}_{{Q}_{yy}}^{2}|{H}_{0}\sim{\chi\:}^{2}\left(r\right(a),0),\end{aligned}$$

where $\:r\:$is the redundancy of the model under $\:{H}_{0}$ and $\:r\left(a\right)\:$is the redundancy of the model when the known ambiguity is excluded from the unknown vector. $\:{\Vert v\Vert}_{M}^{2}={v}^{T}{M}^{-1}v$ denotes the squared norm of $\:v$ weighted by $\:{M}^{-1}$. The AR detector test statistic can be written as (Teunissen 2024)

19 $$\:AR:{\Vert\check{\underline{e}}\Vert}_{{Q}_{yy}}^{2}={\Vert\hat{\underline{e}}\Vert}_{{Q}_{yy}}^{2}+{\Vert\check{\underline{\epsilon\:}}\Vert}_{{Q}_{\hat{a}\hat{a}}}^{2},$$

where $\:\check{\underline{\epsilon}}=\hat{\underline{a}}-\check{\underline{a}}$ is the ambiguity residual. The probability density function (PDF) of $\:\check{\underline{\epsilon}}$ is written as (Teunissen 2002)

20 $$\:{f}_{\check{\underline{\epsilon}}}\left(x\right)=\frac{{\sum}_{z\in{\mathbb{Z}}^{n}}\text{e}\text{x}\text{p}\left\{-\frac{1}{2}{\Vert x+z\Vert}_{{Q}_{\hat{a}\hat{a}}}^{2}\right\}}{\sqrt{\left|2\pi{Q}_{\hat{a}\hat{a}}\right|}}{s}_{0}\left(x\right),$$

with $\:{s}_{0}\left(x\right)$ the indicator function of the integer estimators’ pull-in region of zero vector (Teunissen 2017). With these test statistics, the detectors then read.

21 $$\begin{aligned}&AF:\text{Reject}\:{H}_{0}\:\text{if}\:{\Vert\hat{e}\Vert}_{{Q}_{yy}}^{2}>{\chi}_{\alpha}^{2}\left(r,0\right),\\&AK:\text{Reject}\:{H}_{0}\,\text{if}\,{\Vert\hat{e}\left(a\right)\Vert}_{{Q}_{yy}}^{2}>{\chi}_{\alpha}^{2}\left(r\right(a),0),\\&AR:\text{Reject}\,\text{if}\,{\Vert\check{e}\Vert}_{{Q}_{yy}}^{2}>{\kappa}_{\alpha},\end{aligned}$$

where $\:\alpha\:$ is the given level of significance and $\:{\chi}_{\alpha}^{2}(r,0)$ refers to the $\:1-\alpha\:$ quantile of the central $\:{\chi}^{2}$ PDF with $\:r$ degrees of freedom. The AR critical value $\:{\kappa}_{\alpha}$ cannot be obtained analytically due to the irregular distribution of the AR test statistic; the method to obtain $\:{\kappa}_{\alpha}$ will be introduced in the following section.

The model under $\:{H}_{a}$ is used to evaluate the performance of the detectors for a specific model misspecification, modeled by the additional term $\:Cc$. The distributions of the AF and AK test statistics under $\:{H}_{a}\:$are written as

22 $$\:\begin{aligned} &AF:\:{\Vert\hat{\underline{e}}\Vert}_{{Q}_{yy}}^{2}|{H}_{a}\sim{\chi}^{2}(r,{\lambda}_{\hat{e}}),\\&AK:{\Vert\hat{\underline{e}}\left(a\right)\Vert}_{{Q}_{yy}}^{2}|{H}_{a}\sim{\chi}^{2}\left(r\right(a),{\lambda}_{\hat{e}\left(a\right)}),\end{aligned}$$

with the noncentrality parameters

$$\:{\lambda}_{\hat{e}}={\Vert{P}_{[A,B]}^{\perp}Cc\Vert}_{{Q}_{yy}}^{2},\:\:{\lambda}_{\hat{e}\left(a\right)}={\Vert{P}_{B}^{\perp}Cc\Vert}_{{Q}_{yy}}^{2}.$$

**Fig. 1:**
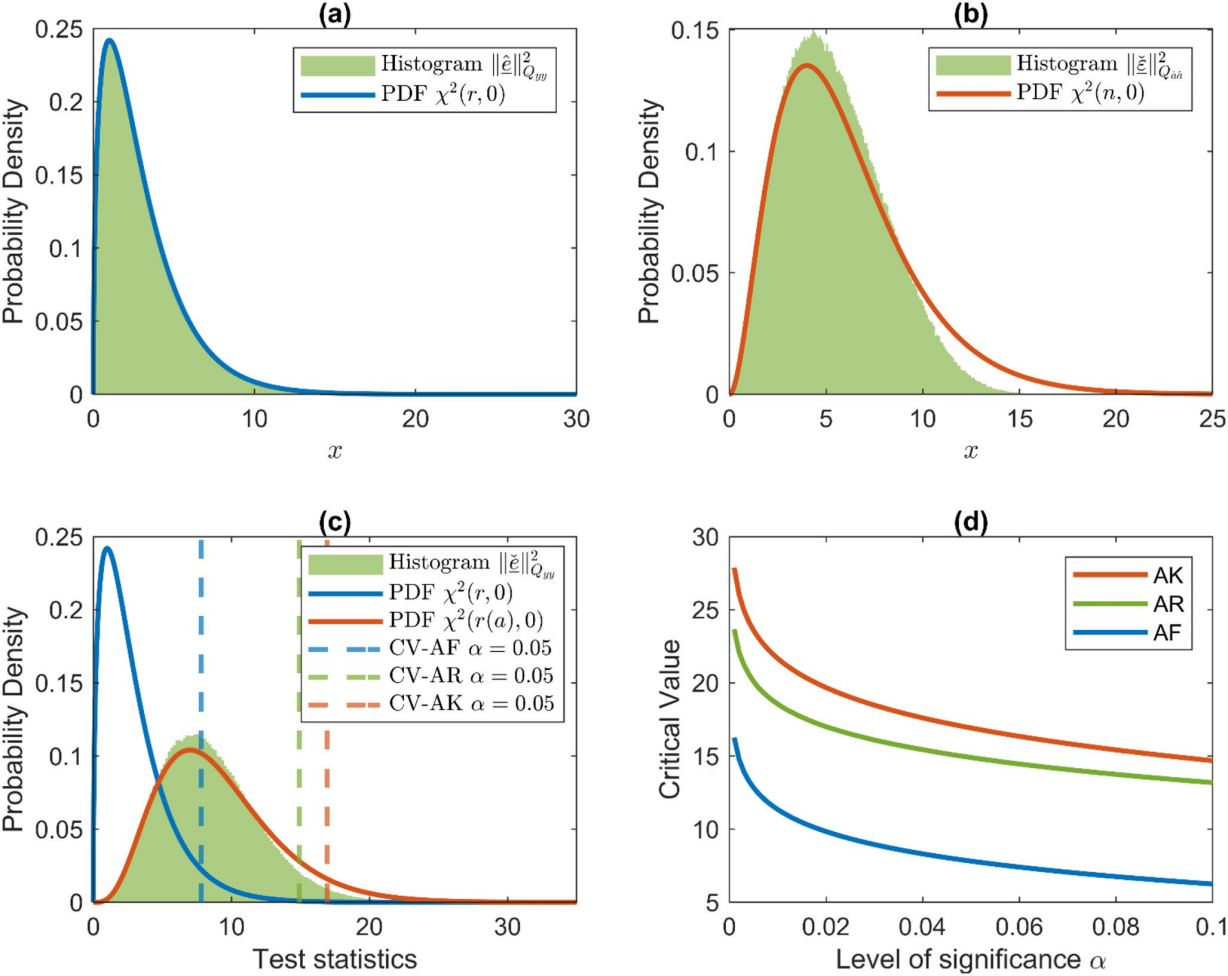
(**a**) Normalized histogram of $\:{\Vert\hat{\underline{e}}\Vert}_{{Q}_{yy}}^{2}$ samples under $\:{H}_{0}$, and $\:{\chi}^{2}(r,0)$ PDF.(**b**) Normalized histogram of $\:{\Vert\check{\underline{\epsilon}}\Vert}_{{Q}_{\hat{a}\hat{a}}}^{2}$ samples under $\:{H}_{0}$, and $\:{\chi}^{2}(n,0)$ PDF. (**c**) Normalized histogram of AR test statistics and PDFs of AR and AK test statistics; dashed lines give critical values of detectors for $\:\alpha=0.05$. (**d**) Critical values of detectors for different $\:\alpha\:$

The distribution of AR test statistic under $\:{H}_{a}$ cannot be written in a closed form; an example is shown in Fig. 1.

## Performance evaluation

### Implementation of detectors

The critical values of the AF and AK detectors can be obtained with the CDF of the central chi-squared distribution with degrees of freedom of $\:r$ and $\:r\left(a\right)$, denoted as $\:{\chi}_{\alpha}^{2}(r,0)$, and $\:{\chi}_{\alpha}^{2}\left(r\right(a),0)$, respectively. The critical value of the AR detector $\:{\kappa}_{\alpha}$ fulfills

23 $$\:{\int}_{0}^{{\kappa}_{\alpha}}{f}_{{\Vert\check{\underline{e}}\Vert}_{{Q}_{yy}}^{2}|{H}_{0}}\left(x\right)dx=1-{\alpha}.$$

Due to the complexity of the distribution of $\:{\Vert\check{\underline{e}}\Vert}_{{Q}_{yy}}^{2}$, we obtain $\:{\kappa}_{\alpha}$ through Monte Carlo simulation (Metropolis and Ulam 1949). $\:{\Vert\check{\underline{e}}\Vert}_{{Q}_{yy}}^{2}\:$can be represented as a sum of $\:{\Vert\hat{\underline{e}}\Vert}_{{Q}_{yy}}^{2}$ and the $\:{\Vert\check{\underline{\epsilon}}\Vert}_{{Q}_{\hat{a}\hat{a}}}^{2}$ (19). Since $\:{\Vert\hat{\underline{e}}\Vert}_{{Q}_{yy}}^{2}\:\text{and}\:{\Vert\check{\underline{\epsilon}}\Vert}_{{Q}_{\hat{a}\hat{a}}}^{2}$ are independent, we can first generate samples of them separately and then obtain samples of $\:{\Vert\check{\underline{e}}\Vert}_{{Q}_{yy}}^{2}$. According to the ‘remove-restore’ property of the integer estimator (Teunissen 1999b), samples of the ambiguity residual can be obtained without knowing the exact value of the true integer ambiguity. We define the number of samples to be generated as $\:{N}_{c}$ and carry out the Monte Carlo simulation using the following steps.

1. Generate *N*_*c*_ samples of the float ambiguity vector, $\:{\hat{a}}_{0}^{\left(1\right)},\:\cdots,\:{\hat{a}}_{0}^{\left(i\right)},\:\cdots,\:{\hat{a}}_{0}^{\left({N}_{c}\right)}$, that follow a normal distribution according to $\:{\mathcal{N}}_{n}\left(0,{Q}_{\hat{a}\hat{a}}\right)$, which denotes an n-dimensional normal distribution with zero mean and variance-covariance matrix $\:{Q}_{\hat{a}\hat{a}}$.
2. Conduct ambiguity resolution (Teunissen 1999a) to obtain samples of the resolved ambiguity, $\:{\check{a}}_{0}^{\left(1\right)},\:\cdots,\:{\check{a}}_{0}^{\left(i\right)},\:\cdots,\:{\check{a}}_{0}^{\left({N}_{c}\right)}$. Then samples of $\:{\Vert\check{\underline{\epsilon}}\Vert}_{{Q}_{\hat{a}\hat{a}}}^{2}$ can be obtained with $\:{\Vert{\check{\epsilon}}_{0}\Vert}_{{Q}_{\hat{a}\hat{a}}}^{2,\left(i\right)}={\Vert{\hat{a}}_{0}^{\left(i\right)}-{\check{a}}_{0}^{\left(i\right)}\Vert}_{{Q}_{\hat{a}\hat{a}}}^{2}$.
3. Generate *N*_*c*_ samples of the AF residual, $\:{\Vert{\hat{e}}_{0}\Vert}_{{Q}_{yy}}^{2,\left(1\right)},\:\cdots,\:{\Vert{\hat{e}}_{0}\Vert}_{{Q}_{yy}}^{2,\left(i\right)},\:\cdots,\:{\Vert{\hat{e}}_{0}\Vert}_{{Q}_{yy}}^{2,\left({N}_{c}\right)}$, that follow a $\:{\chi}^{2}(r,0)$ distribution.
4. Obtain samples of the AR test statistic with $\:{\Vert{\check{e}}_{0}\Vert}_{{Q}_{yy}}^{2,\left(i\right)}={\Vert{\hat{e}}_{0}\Vert}_{{Q}_{yy}}^{2,\left(i\right)}+{\Vert{\check{\epsilon}}_{0}\Vert}_{{Q}_{\hat{a}\hat{a}}}^{2,\left(i\right)}.$
5. Finally, sort the samples $\:{\Vert{\check{e}}_{0}\Vert}_{{Q}_{yy}}^{2,\left(i\right)}$ in ascending order, the critical value is approximated by the $\:\left[\left(1-\alpha\right){N}_{c}\right]$-th ordered sample, $\:\left[\cdot\right]\:$denotes the rounding operation.

The number of samples *N*_*c*_ determines the precision of the critical value simulation. It can be chosen according to Yin et al. (2024), where the relation between *N*_*c*_ and simulation uncertainty is investigated.

### Uncertainty of the AR critical value

The procedure to obtain the AR critical value falls under the topic of Monte Carlo quantile simulation, as the unknown critical value is the $\:1-\alpha$ quantile of the PDF $\:{f}_{{\Vert\check{\underline{e}}\Vert}_{{Q}_{yy}}^{2}|{H}_{0}}\left(x\right)$, and it is approximated by the $\:\left[\left(1-\alpha\right){N}_{c}\right]$-th ordered sample. The distributional property of the ordered sample and the quantile simulated with the Monte Carlo method are introduced by Serfling (1980), where several approaches to evaluate the uncertainty of the quantile simulation are also provided. We describe one of the approaches based on asymptotic normality.

It is shown by Serfling (1980) that the simulated $\:1-\alpha$ quantile $\:{\hat{\underline{\kappa}}}_{\alpha}$ with *N*_*c*_ samples follows

24 $$\begin{aligned}&\sqrt{{N}_{c}\:}\left({\hat{\underline{\kappa}}}_{\alpha}-{\kappa}_{\alpha}\right)\to{\mathcal{N}}_{1}\left(0,{\sigma}^{2}\right),\:\text{when}\:{N}_{c}\to\infty,\\&\:\text{with}\:{\sigma}^{2}=\frac{\alpha\left(1-\alpha\right)}{{f}_{{\Vert\check{\underline{e}}\Vert}_{{Q}_{yy}}^{2}|{H}_{0}}^{2}\left({\kappa}_{\alpha}\right)}\end{aligned}$$

in which $\:{\kappa}_{\alpha}$ denotes the true but unknown critical value. This property provides the asymptotic variance $\:{\sigma}^{2}$ and indicates that $\:{\hat{\underline{\kappa}}}_{\alpha}$ is asymptotically consistent. Although $\:{f}_{{\Vert\check{\underline{e}}\Vert}_{{Q}_{yy}}^{2}|{H}_{0}}\left({\kappa}_{\alpha}\right)$ is unknown, it can be approximated by the probability density around $\:{\hat{\kappa}}_{\alpha}$ obtained based on the samples. Then $\:{\sigma}^{2}/{N}_{c}$ approximates the variance of the simulated critical value. A detailed example of this method can be found in (Yin et al. 2024).

## Statistical power of detectors

We evaluate the performance of the AF, AK, and AR detectors in the following way:

- We first select a misspecification, which is modeled by the term $\:Cc$ under $\:{H}_{a}$.
- Then, we obtain the statistical power of the AK, AF, and AR detector to detect the selected misspecification for a particular size $\:c$ and level of significance $\:\alpha\:$;
- We compare the performance of the detectors by comparing the power.

Under $\:{H}_{a},\:$ AF and AK test statistics follow non-central chi-squared distributions (22), and the power to detect a selected model misspecification with size $\:c$ can be obtained with the CDF of their distributions as

25 $$\:\begin{aligned}&{\gamma}_{AF}=P\left[{\Vert\hat{\underline{e}}\Vert}_{{Q}_{yy}}^{2}>{\chi}_{\alpha}^{2}(r,0)|{H}_{a}\right],\\&{\gamma}_{AK}=P\left[{\Vert\hat{\underline{e}}\left(a\right)\Vert}_{{Q}_{yy}}^{2}>{\chi}_{\alpha}^{2}\left(r\right(a),0)|{H}_{a}\right].\end{aligned}$$

We obtain now the power of the ambiguity-resolved detector by Monte Carlo simulation. The mean of $\:\hat{\underline{a}}$ under $\:{H}_{a}$ is

26 $$\:E\left\{\hat{\underline{a}}\right|{H}_{a}\}=a+{\bar{A}}^{+}Cc$$

and the noncentrality parameter of $\:{\Vert\underline{\hat{e}}\Vert}_{{Q}_{yy}}^{2}$ is computed according to (22). Similar to the critical value simulation, we generate samples of $\:{\Vert\hat{\underline{e}}\Vert}_{{Q}_{yy}}^{2}\:\text{a}\text{n}\text{d}\:{\Vert\check{\underline{\epsilon}}\Vert}_{{Q}_{\hat{a}\hat{a}}}^{2}$ independently with $\:a=0$. We simulate the power using $\:{N}_{p}\:$samples as follows.

1. Generate *N*_*p*_ samples of the float ambiguity vector $\:{\hat{a}}_{a}^{\left(1\right)},\:\cdots,\:{\hat{a}}_{a}^{\left(i\right)},\:\cdots,\:{\hat{a}}_{a}^{\left({N}_{p}\right)}$, which follow a normal distribution according to $\:{\mathcal{N}}_{n}\left(E\right\{\hat{\underline{a}}\left|{H}_{a}\right\},\:{Q}_{\hat{a}\hat{a}})$.
2. Conduct ambiguity resolution (Teunissen 1999a) to obtain samples of the resolved ambiguity $\:{\check{a}}_{a}^{\left(1\right)},\:\cdots,\:{\check{a}}_{a}^{\left(i\right)},\:\cdots,\:{\check{a}}_{a}^{\left({N}_{p}\right)}$. Then samples of $\:{\Vert\check{\underline{\epsilon}}\Vert}_{{Q}_{\hat{a}\hat{a}}}^{2}$ can be obtained with $\:{\Vert{\check{\epsilon}}_{a}\Vert}_{{Q}_{\hat{a}\hat{a}}}^{2,\left(i\right)}={\Vert{\hat{a}}_{a}^{\left(i\right)}-{\check{a}}_{a}^{\left(i\right)}\Vert}_{{Q}_{\hat{a}\hat{a}}}^{2}.$
3. Generate *N*_*p*_ samples of the AF residual $\:{\Vert{\hat{e}}_{a}\Vert}_{{Q}_{yy}}^{2,\left(1\right)},\:\cdots,\:{\Vert{\hat{e}}_{a}\Vert}_{{Q}_{yy}}^{2,\left(i\right)},\:\cdots,\:{\Vert{\hat{e}}_{a}\Vert}_{{Q}_{yy}}^{2,\left({N}_{p}\right)}$, that follow a $\:{\chi}^{2}(r,{\lambda}_{\hat{e}})$ distribution (22).
4. Samples of the AR test statistic can be computed as $\:{\Vert{\check{e}}_{a}\Vert}_{{Q}_{yy}}^{2,\left(i\right)}={\Vert{\hat{e}}_{a}\Vert}_{{Q}_{yy}}^{2,\left(i\right)}+{\Vert{\check{\epsilon}}_{a}\Vert}_{{Q}_{\hat{a}\hat{a}}}^{2,\left(i\right)}$.
5. Finally, count the number of samples $\:{\Vert{\check{e}}_{a}\Vert}_{{Q}_{yy}}^{2,\left(i\right)}$ larger than the critical value, denoted as $\:{n}_{p}$. The simulated power in percentage follows as $\:\hat{\gamma}\left(c\right)=100\times\frac{{n}_{p}}{{N}_{p}}\%\:$.

## Uncertainty of AR detection power

The power of the AR detector is obtained by a two-stage Monte Carlo simulation procedure. We evaluate the uncertainty of the power simulation empirically by repeating the simulation for $\:{N}_{r}$ times (Morio and Balesdent 2015).

1. Assume we use a total number of $\:{N}_{1}$ samples to simulate the critical value and $\:{N}_{2}$ samples to simulate the power. We conduct $\:{N}_{r}$ repeated simulations, and each time with $\:{N}_{c}={N}_{1}/{N}_{r}\:$for the critical value and $\:{N}_{p}={N}_{2}/{N}_{r}$ for the power. $\:{N}_{r}$ can be chosen as 50 or 100 in practice (Morio and Balesdent 2015; El Masri et al. 2021).
2. Simulate the critical value and the power for the selected misspecification with size *c*. Repeat this step $\:{N}_{r}\:$times and get $\:{N}_{r}\:$power simulations denoted as $\:{\hat{\gamma\:}}_{i}\left(c\right), i=\:1, \:2, \:\cdots, \:{N}_{r}$.
3. The mean of $\:{N}_{r}$ power simulations is the final simulated power,

27 $$\:\hat{\gamma}\left(c\right)=\frac{1}{{N}_{r}}\sum_{i=1}^{{N}_{r}}{\hat{\gamma}}_{i}\left(c\right).$$

Its standard deviation is computed assuming $\:{\hat{\gamma}}_{i}\left(c\right)$ are independent and of the same precision,

28 $$\:{\sigma}_{\hat{\gamma}\left(c\right)}=\sqrt{\frac{1}{{N}_{r}}{\sigma}_{{\hat{\gamma}}_{i}\left(c\right)}},\:\text{w}\text{i}\text{t}\text{h}\:{\sigma}_{{\hat{\gamma}}_{i}\left(c\right)}=\sqrt{\frac{{\sum}_{i=1}^{{N}_{r}}{\left({\hat{\gamma}}_{i}\left(c\right)-\hat{\gamma}\left(c\right)\right)}^{2}}{{N}_{r}-1}}.$$

### Experiments

In this section, simulation experiments are conducted to evaluate the performance of the AR detector. We first consider one-dimensional misspecifications. An experiment with a single-frequency GPS model is carried out, where the distributions of the test statistics under $\:{H}_{0}$ and $\:{H}_{a}$ and the detection power of detectors as functions of misspecifications’ size are presented. Then, the power functions for a dual-frequency GPS model are provided. After that, the performance of the AR detector is evaluated by simulation experiments over 25 user locations and five models with 72 satellite geometries per location per model. Finally, we present three examples of detecting multi-dimensional misspecifications. The experiments for phase outlier detection employ two-epoch models, and single-epoch models are used for the other misspecifications.

### Performance for single-frequency model

**Fig. 2:**
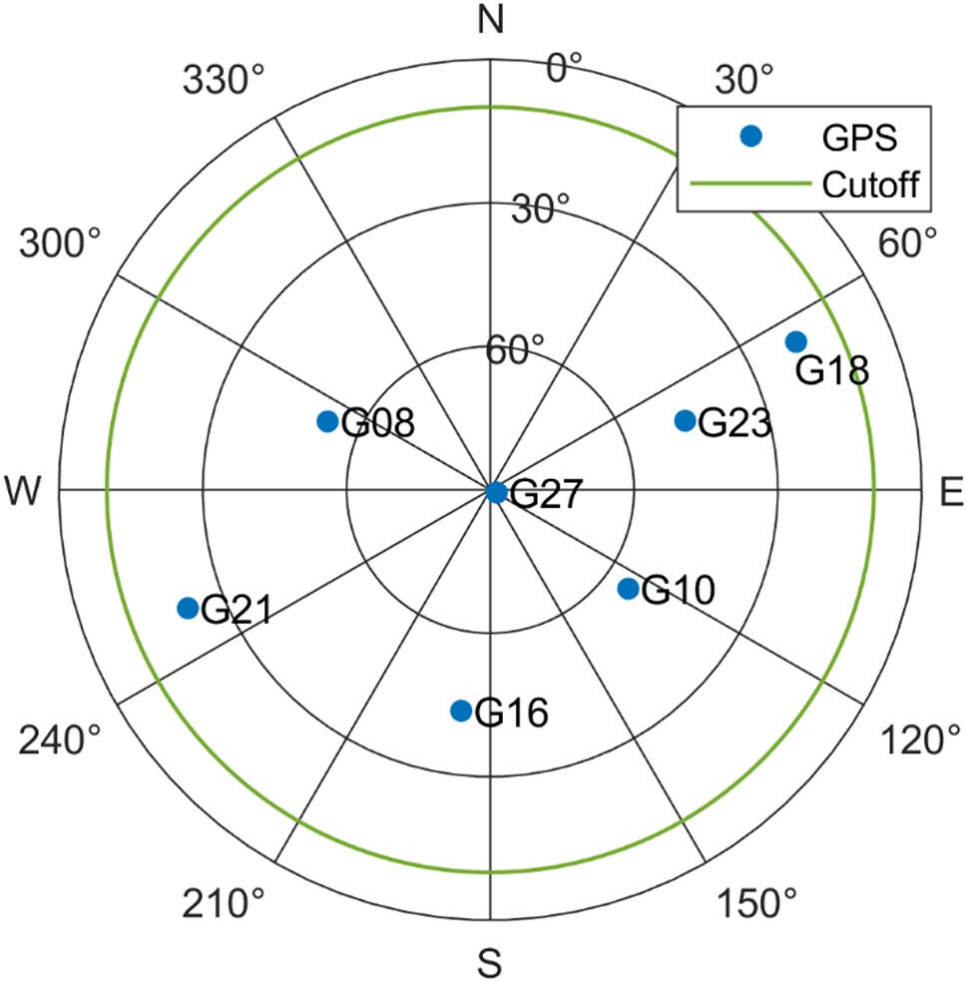
Skyplot of GPS satellites for single-frequency experiment

In this simulation experiment, we consider single-frequency (L1) observables from GPS satellites. Seven satellites are observed, and the skyplot is shown in Fig. 2. To compute $\:{Q}_{yy}$ with (4), the zenith-referenced standard deviations for pseudorange and carrier phase observables are set as $\:{\sigma}_{p}=0.2\text{m}$ and $\:{\sigma}_{\varphi}=0.002\text{m}$, respectively.

### PDFs under *H*_0_

We evaluate the performance of detecting a blunder in pseudorange, a phase outlier, and atmosphere delays with the DD observation model. The ILS success rate (14) of the single-epoch ambiguity resolution is 80.5%. As we describe in ‘Implementation of detectors’, the critical value of the AR detector is obtained by Monte Carlo simulation. The histograms in Fig. 1 are normalized to provide the probability density. We use $\:{10}^{6}$ samples for Monte Carlo simulation with 200 bins in each histogram. Figure 1a presents the distribution $\:{\chi}^{2}(r,0)$ and normalized histogram of $\:{\Vert\hat{\underline{e}}\Vert}_{{Q}_{yy}}^{2}$ samples under $\:{H}_{0}$. Figure 1b compares the normalized histogram of $\:{\Vert\check{\underline{\epsilon}}\Vert}_{{Q}_{\hat{a}\hat{a}}}^{2}$samples under $\:{H}_{0}$, and the PDF of $\:{\chi}^{2}(n,0)$, which is the distribution of $\:{\Vert\hat{\underline{\epsilon}}\left(a\right)\Vert}_{{Q}_{\hat{a}\hat{a}}}^{2}$ with the $\:n$ dimensional known ambiguity vector. $\:\check{\underline{\epsilon}}$ is always bounded inside the pull-in region of the integer estimator; thus, its square norm is also bounded. As a result, the normalized histogram of $\:{\Vert\check{\underline{\epsilon}}\Vert}_{{Q}_{\hat{a}\hat{a}}}^{2}$ samples is compressed along the horizontal axis compared with $\:{\chi}^{2}(n,0).$ Fig. 1c exhibits the distributions of three detectors. The AF and AK test statistics follow chi-squared distributions (18), and the distribution of the AR test statistic lies between them. The reason is that the AR test statistic is compressed compared with the AK test statistic, as shown in Fig. 1b, and is larger than the AF test statistic due to including the square norm of ambiguity residual. Figure 1d presents the critical values of the three detectors for different levels of significance.

**Table 1:** Detection power corresponding to Fig. 3 ($\:{\alpha}=0.05$)

Misspecification	Size ($\:{c}$)	AF	AR	AK
Blunder in pseudorange (G08)	1.5 m	59%	79%	92%
Phase outlier (G08)	0.03 m	68%	92%	100%
Ionosphere delay (G08)	0.27 TECU	5%	26%	100%
Troposphere delay	0.07 m	5%	24%	100%

**Fig. 3:**
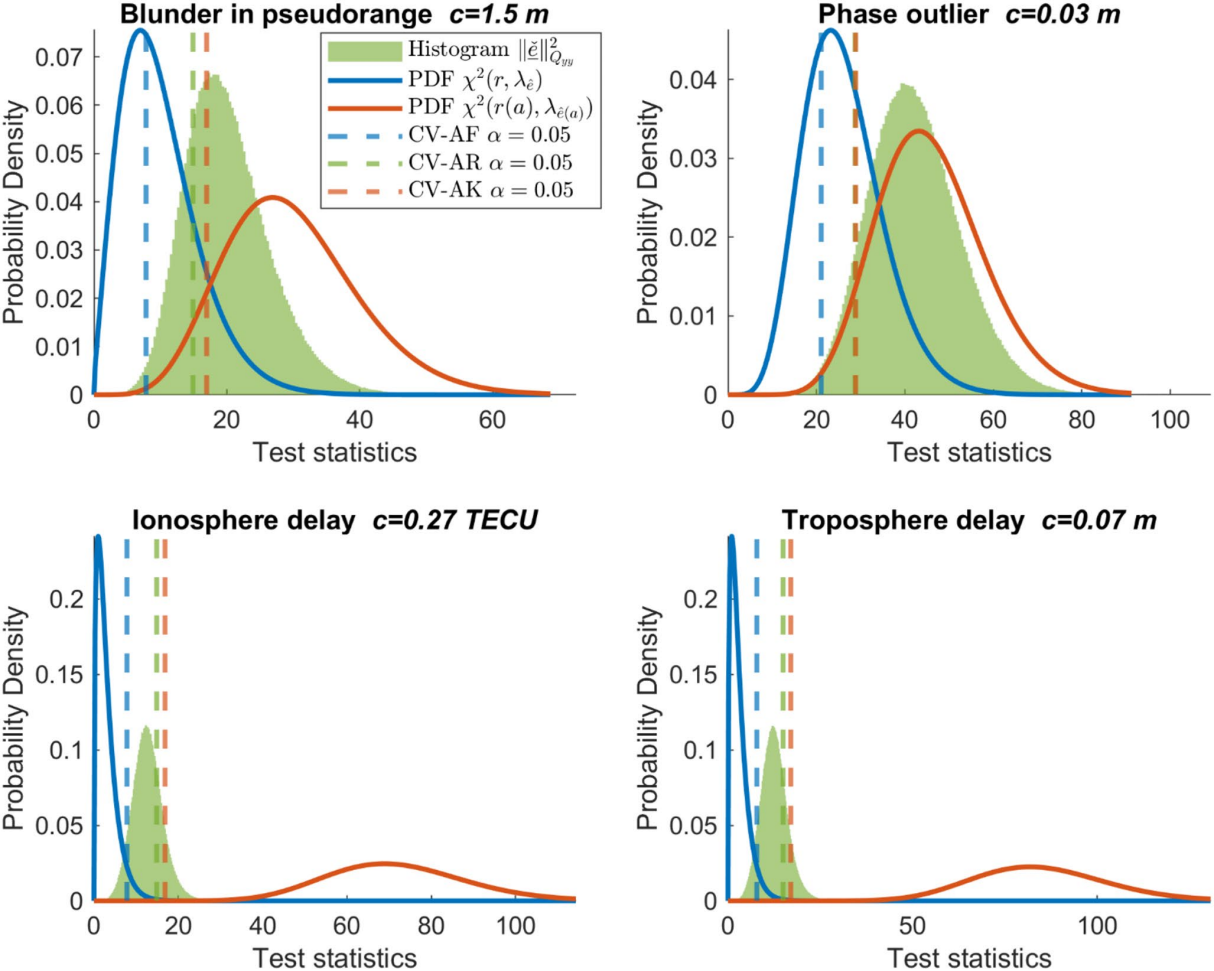
Distributions of three test statistics under $\:{H}_{a}$ for four one-dimensional misspecifications. Blue and red lines are distributions of AF and AK test statistics. Normalized histogram in green illustrates AR distribution. Dashed lines give their critical values for $\:\alpha\:=0.05$

### PDFs under *H*_*a*_

**Fig. 4:**
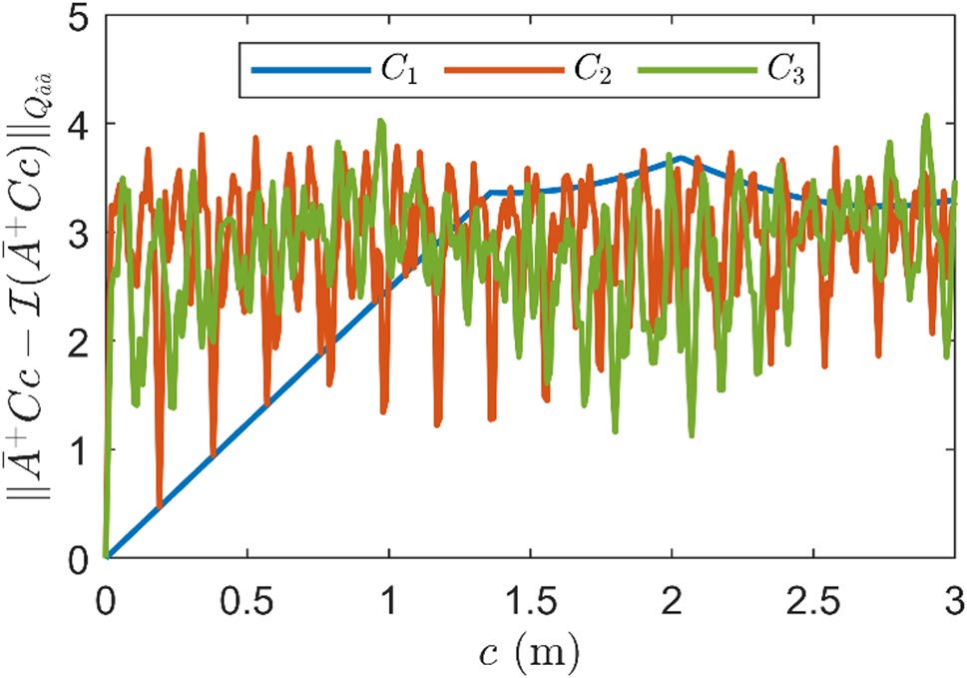
Distance (under metric $\:{Q}_{\hat{a}\hat{a}}$) between $\:{\bar{A}}^{+}Cc$ and nearest integer of it, denoted by $\:\mathcal{I}\left({\bar{A}}^{+}Cc\right)$, as functions of misspecifications’ size for a blunder in pseudorange ($\:{C}_{1}$), ionosphere delay ($\:{C}_{2}$), and troposphere delay ($\:{C}_{3}$)

Figure 3 shows the distributions of the test statistics under $\:{H}_{a}$ for four one-dimensional misspecifications, and the corresponding detection powers are given in Table 1. Compared with the distributions under $\:{H}_{0}$ (Fig. 1c), the high-density regions of the distributions under $\:{H}_{a}$ are shifted toward the larger values. This is due to the noncentrality introduced by the misspecifications to the AF and AK test statistics. For the AR test statistic, the change in its distribution combines the noncentrality of $\:{\Vert\hat{\underline{e}}\Vert}_{{Q}_{yy}}^{2}|{H}_{a}$ and the bias of $\:\hat{\underline{a}}|{H}_{a}$ that affects the distribution of $\:{\Vert\check{\underline{\epsilon}}\Vert}_{{Q}_{\hat{a}\hat{a}}}^{2}|{H}_{a}$. The impact of $\:{\Vert\check{\underline{\epsilon}}\Vert}_{{Q}_{\hat{a}\hat{a}}}^{2}$ will be further explained in Fig. 4. The detection power is the probability that a misspecification (with size $\:c$) can be detected. For a blunder in the pseudorange of satellite G08 with the size of 1.5 m, the AR detector increases the power by 20%. The phase outlier of satellite G08 with 3 cm can be detected with a 92% power by the AR detector, which is higher than the 68% float detection power. The AF detector can hardly detect the differential atmosphere delays of several centimeters in this experiment. However, once the ambiguity is known, the AK detector can detect them with a power equal to one. This can be explained by the closed form of the AF and AK noncentrality parameters (Eqs. 22 and 23 in Teunissen 2024). Once the ambiguity is known, the noncentrality parameters of the ionosphere and troposphere delays are magnified by the factor $\:{\sigma}_{p}^{2}/{\sigma}_{\phi}^{2}$, which equals 10,000 in this experiment and drives the difference in the detection power of AF and AK detectors. Although the single-epoch DD ambiguity resolution success rate in this experiment is only 80.5%, we still observe the improvement in the detection power of the AR detector compared with the AF detector. Table 1 also shows the significant differences between the power of the AR and AK detectors. Since the ambiguities are unknown in practice even when they are integer-estimated, one should not predict the performance of the AR detector relying on the assumed probabilistic property of the AK detector.

### Power functions

**Fig. 5:**
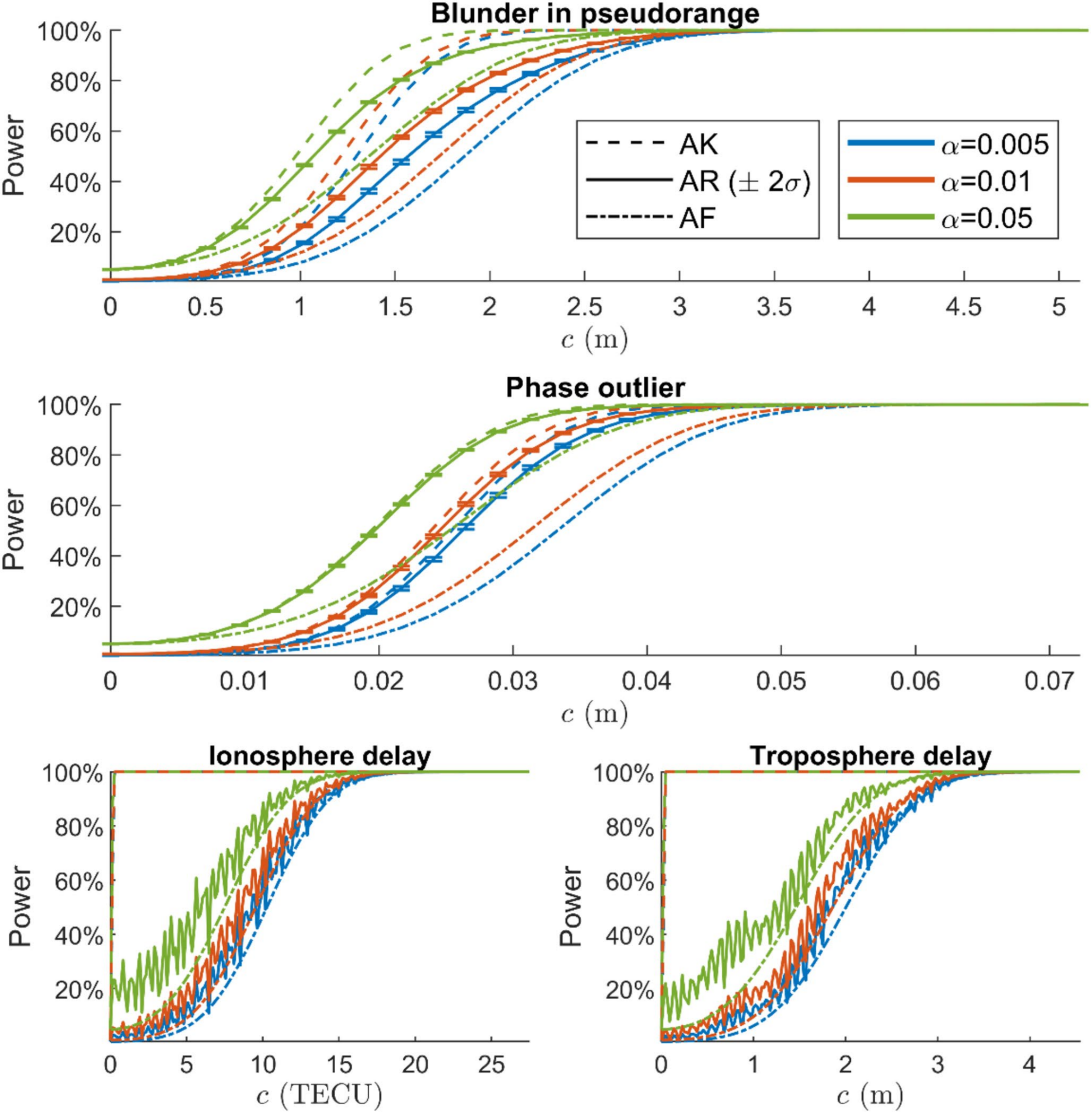
Detection power for blunder in pseudorange (G08), phase outlier (G08), ionosphere (G08), and troposphere as functions of misspecifications’ size with single-frequency model. The uncertainty of simulated AR power is shown by $\:\pm\:2\sigma\:$ vertical error bars in first and second graphs

Figure 5 presents the power of the AF, AK, and AR detectors as functions of four misspecifications’ sizes evaluated with the single frequency model. The vertical error bars with $\:\pm\:2\sigma\:$ for the simulated AR powers are plotted for the blunder in pseudorange and phase outlier power functions. The power and uncertainty are obtained with the steps described in ‘Uncertainty of AR detection power’ with $\:{N}_{1}={N}_{2}=2\times\:{10}^{5}$ and $\:{N}_{r}=50$. The powers are evaluated for 31 $\:c$ values for the blunder in pseudorange and phase outlier, and 201 $\:c$ values for the atmosphere delays.

The AF and AK power functions are smooth curves since the noncentrality of their test statistics monotonically increases with $\:c$. However, as observed in the atmosphere delay experiments, the AR power function can be non-monotonic, which can be explained as follows. The AR power can be written as (Teunissen 2024)

29 $$\:{\gamma}_{AR}=\int P\left[{\Vert\check{\underline{\epsilon}}\Vert}_{{Q}_{\hat{a}\hat{a}}}^{2}>{\kappa}_{\alpha}-x|{H}_{a} \right] {f}_{\underline{x}}\left(x|{H}_{a} \right) dx,$$

where $\:\underline{x}={\Vert\hat{\underline{e}}\Vert}_{{Q}_{yy}}^{2}$. With this formulation, the impact of $\:Cc$ on $\:{\Vert\hat{\underline{e}}\Vert}_{{Q}_{yy}}^{2}$ is captured by $\:{f}_{\underline{x}}\left(x\right|{H}_{a})$ and its impact on $\:{\Vert\check{\underline{\epsilon}}\Vert}_{{Q}_{\hat{a}\hat{a}}}^{2}$ is captured by the probability inside the integral. Although the noncentrality of $\:{\Vert\hat{\underline{e}}\Vert}_{{Q}_{yy}}^{2}$ monotonically increases with $\:c$, this is not the case for the probability $\:P[{\Vert\check{\underline{\epsilon}}\Vert}_{{Q}_{\hat{a}\hat{a}}}^{2}>{\kappa}_{\alpha}-x|{H}_{a}]$. This probability will be small if the bias of $\:\hat{\underline{a}}|{H}_{a},\:\text{w}\text{h}\text{i}\text{c}\text{h}\:\text{e}\text{q}\text{u}\text{a}\text{l}\text{s}\:{\bar{A}}^{+}Cc$, is close to an integer, and it will be larger otherwise. Figure 4 presents the distance between $\:{\bar{A}}^{+}Cc$ and the nearest integer as functions of $\:c$ for the blunder in pseudorange ($\:{C}_{1}$), ionosphere delay ($\:{C}_{2}$), and troposphere delay ($\:{C}_{3}$). For atmosphere delays, the distance oscillates with $\:c$, while it increases smoothly for the pseudorange. The explanation for this behaviour is provided by Fig. 8 in (Teunissen 2024).

**Fig. 6:**
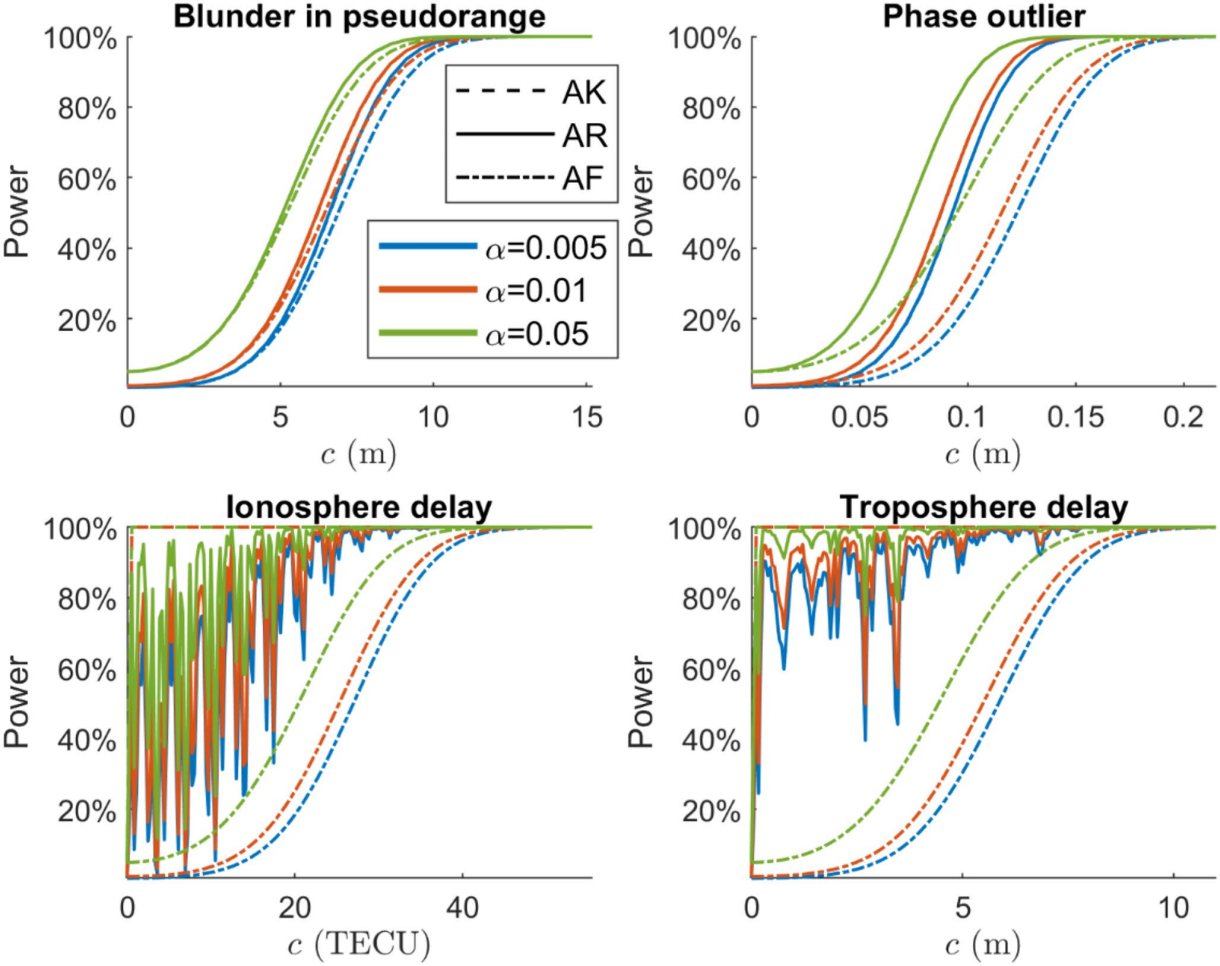
Power functions of detecting blunder in pseudorange (G05), phase outlier (G05), ionosphere (G05) and troposphere delays with dual-frequency GPS model

In almost all cases, the AR power functions lie between the AK and AF power functions. This means the AR detector provides a larger detection power than the AF detector for detecting the same misspecification. However, the AR detector may provide a lower power than the AF detector in an exceptional case where the bias $\:{\bar{A}}^{+}Cc$ is close to an integer. In this case, the additional term in the AR test statistic $\:{\Vert\check{\underline{\epsilon}}\Vert}_{{Q}_{\hat{a}\hat{a}}}^{2}$ turns out to be small and does not contribute to the detection of the misspecification. This case rarely happens for several $\:c$ values, as is shown in the power functions of the atmosphere delays in Fig. 5.

**Fig. 7:**
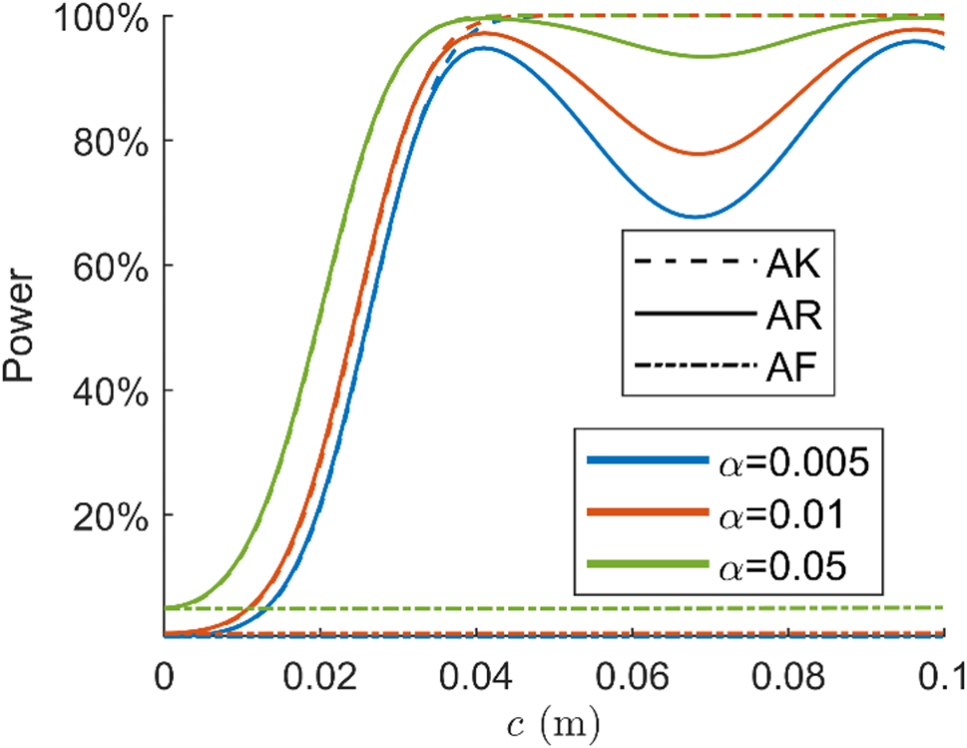
Power functions of constant bias detection for single-frequency SD model

The power functions of the constant bias detection for the SD common oscillator model are presented in Fig. 7. The powers are evaluated for 101 $\:c$ values. The ambiguity resolution success rate for this experiment is $\:99.3\%$, which is higher than that of the DD ambiguity since the between-satellite difference is not conducted. The receiver clock offsets and hardware delays are assumed to be canceled by the between-receiver differencing in the model under $\:{H}_{0}$. The detection power is evaluated for $\:c$ in the range of 0 to 0.1 m since the constant bias is several centimeters, as shown by Keong (1999). The constant bias belongs to the ‘tropo-type’ misspecifications described by Teunissen (2024), which affects the pseudorange and the carrier phase identically. The noncentrality parameter of the AK detector is $\:{\sigma}_{p}^{2}/{\sigma}_{\phi}^{2}$ times larger than that of the AF detector. As is shown by the power functions, the AK detection power is close to 100% for $\:c$ larger than 4 cm, while the AF detector is insensitive to the centimeter-level bias. The AR detection power is very close to that of the AK detector for the bias smaller than 4 cm. When bias increases, we observe a non-monotonic behavior that is driven by the distribution of $\:{\Vert\check{\underline{\epsilon}}\Vert}_{{Q}_{\hat{a}\hat{a}}}^{2}$.

### Performance for dual-frequency model

**Fig. 8:**
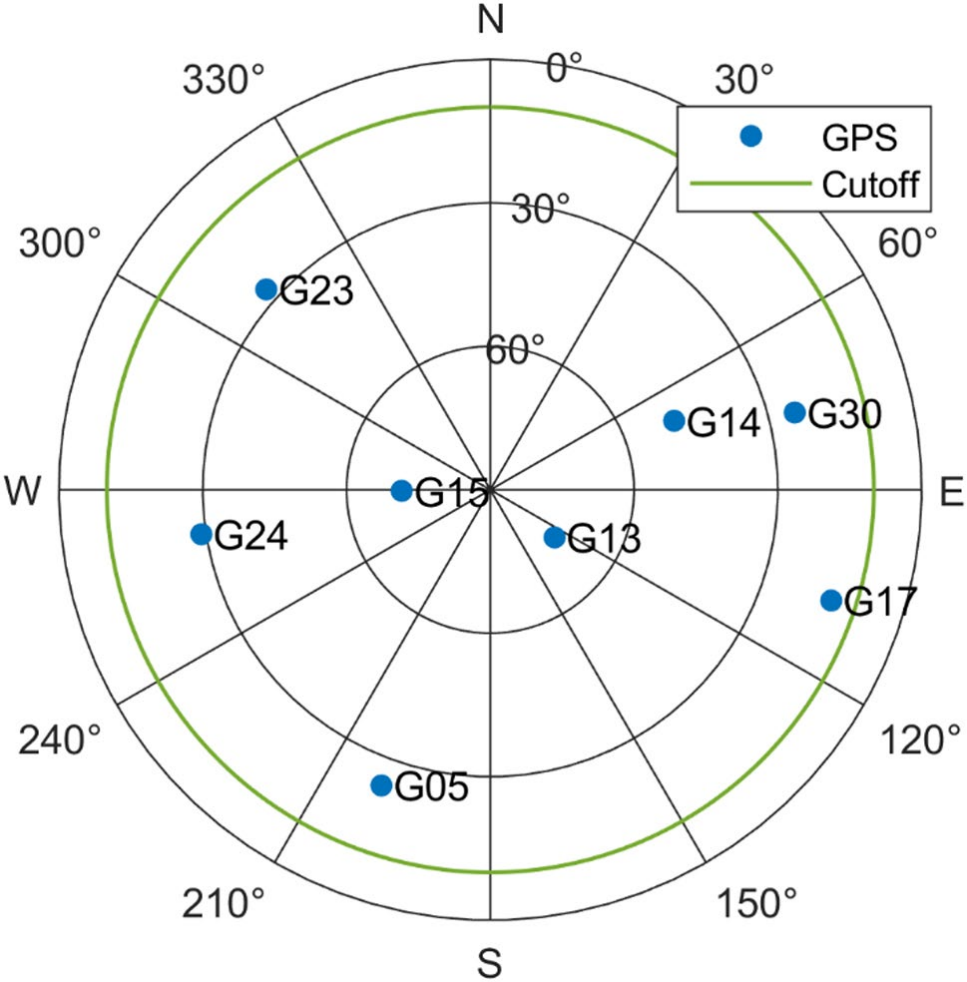
Skyplot of GPS satellites for dual-frequency experiment

In this simulation experiment, we evaluate the performance of the AR detector with a dual-frequency (L1/L5) GPS observation model, and the skyplot of the satellites is shown in Fig. 8. The zenith-referenced standard deviations for pseudorange and carrier phase observables are set as $\:{\sigma\:}_{p}=0.5\text{m}$ and $\:{\sigma\:}_{\varphi\:}=0.005\text{m}$, with which we assume the observables are collected from a low-cost receiver.

**Fig. 9:**
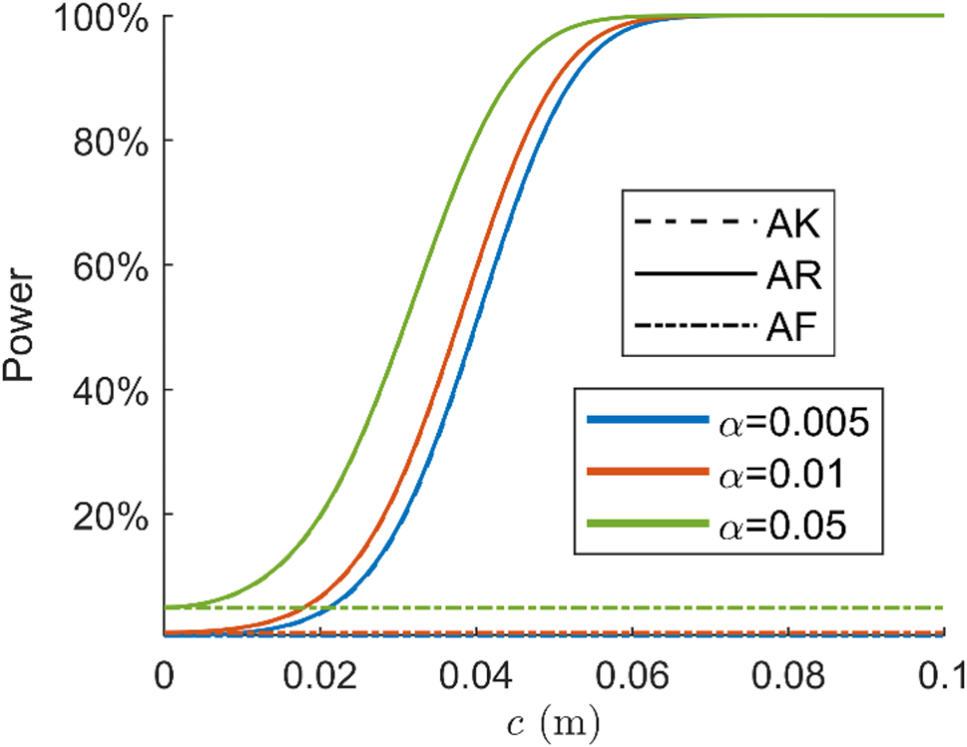
Power functions of SD constant bias detection with dual-frequency model

Figure 6 presents the power of the AF, AK, and AR detectors as functions of four misspecifications’ size for the dual-frequency DD model; the ILS success rate of the single-epoch DD ambiguity resolution is $\:97.4\%$. Figure 9 shows the power functions of the constant bias detection for an SD common oscillator model; the ILS success rate of the SD ambiguity resolution is $\:99.9\%$. The number of samples for the Monte Carlo simulation and the number of $\:c$ values used to compute powers are the same as the previous experiment with the single-frequency model. For detecting the blunder in pseudorange, phase outlier, and constant bias, the AR detector performs almost the same as the AK detector. Similar to Fig. 5, the power functions of the atmosphere delays are oscillating. Compared with the single-frequency experiments, the AR power functions in the dual-frequency case are closer to the AK detector due to the higher success rate.

To provide a quantitative description of the improvement in the AR detection power compared with the AF detector, we compute the *average power difference* between the AR and AF detection power with

30 $$\:\frac{1}{{N}_{d}}\sum_{i=1}^{{N}_{d}}{\gamma}_{AR}\left({c}_{i}\right)-{\gamma}_{AF}\left({c}_{i}\right)$$

where $\:{N}_{d}$ is the number of power points that are taken into account, $\:{c}_{i}$ is the $\:i$-th $\:c$ value for which powers are evaluated. We consider the powers fulfilling $\:10\%<{\gamma\:}_{AR}\left({c}_{i}\right)<90\%$, since the power functions are convergent when the power is close to $\:\alpha$ and 1 (See Table 2).

**Table 2:** Average power difference of dual-frequency experiment

Misspecification	$\:{\alpha}=0.005$	$\:{\alpha}=0.01$	$\:{\alpha}=0.05$
Blunder in pseudorange	5%	4%	2%
Phase outlier	28%	26%	19%
Ionosphere delay	49%	52%	51%
Troposphere delay	72%	73%	57%
Constant Bias	48%	47%	41%

As is shown in Table 2, improvement of the power is limited for detecting the blunder in pseudorange, although the AR detector performs almost the same as the AK detector. This can be explained by the noncentrality parameter for ‘code-type’ misspecifications derived by Teunissen (2024), which shows that the noncentrality parameters of both the AK and AF detectors are driven by pseudorange precision. For detecting the phase outlier, we observe 28% and 26% increases in power on average for $\:\alpha=0.005$ and $\:\alpha=0.01$. The power increases are over 41% and up to 73% on average for detecting the atmosphere delays and constant bias. In this simulation experiment with the dual-frequency GPS observation model, the AR detector performs better than the float detector as it provides higher detection powers.

### Performance evaluation at different locations

**Fig. 10:**
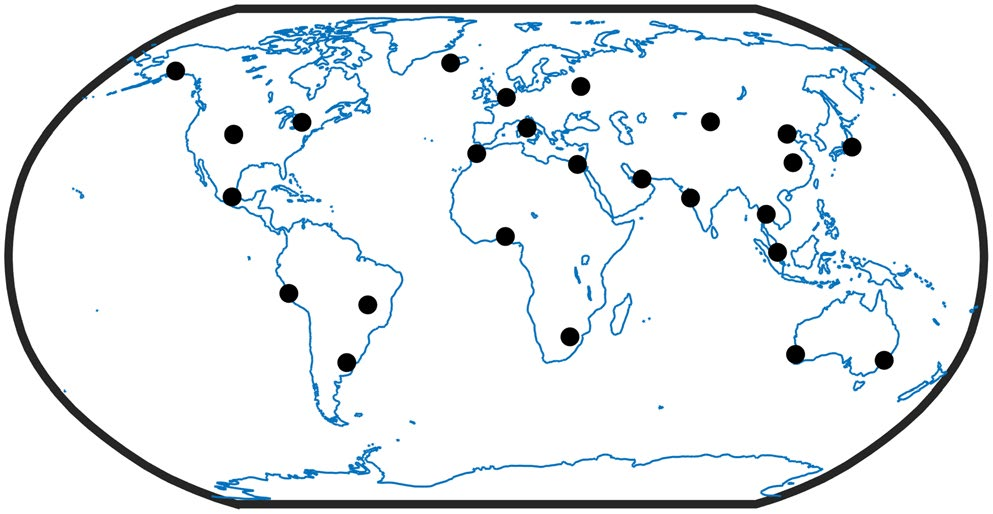
User locations for AR detector performance evaluation

We conduct simulation experiments over 25 user locations, which are shown in Fig. 10, with five types of observation models and obtain the power functions of the AR and AF detectors for four misspecifications: blunder in pseudorange, phase outlier, ionosphere, and troposphere delays. For each user location and model, we formulate the DD observation equations with 72 satellite geometries, obtained every 20 min for 24 h from the precise orbit product of the IGS (International GNSS Service).

We are interested in the performance of the AR detector when the ambiguity resolution success rate of the model is not close to one. Therefore, we conduct experiments with relatively weak models listed in Table 3. We obtain 7200 power functions for detecting a blunder in pseudorange and atmosphere delays and 5400 power functions for phase outlier detection. The average power difference is computed with (30) for all power functions to compare the performance of the AR detector with the AF detector.

**Table 3:** Observation models for AR detector performance evaluation

Misspecifications	Constellation	Signal	Epochs	$\:{{\sigma}}_{{p}}({{\sigma}}_{{\varphi}}={{\sigma}}_{{p}}/100)$
Blunder in pseudorange Ionosphere delay Troposphere delay	GPS	L1	1	{0.2, 0.35}m
	GPS	L1 + L5	1	0.5 m
	GPS + Galileo	L1 + E1	1	0.5 m
Phase outlier	GPS	L1	2	{0.2, 0.35}m
	GPS + Galileo	L1 + E1	2	0.5 m

**Table 4:** Overall average of power difference in four success rate intervals

Misspecifications	$\:[0.775,\:0.8]$	$\:[0.875,\:0.9]$	$\:[0.925,\:0.95]$	$\:[0.975,\:1]$
Blunder in pseudorange	1%	-0%	-1%	-1%
Phase outlier	4%	7%	8%	12%
Ionosphere delay	10%	17%	24%	47%
Troposphere delay	17%	32%	45%	60%

**Fig. 11:**
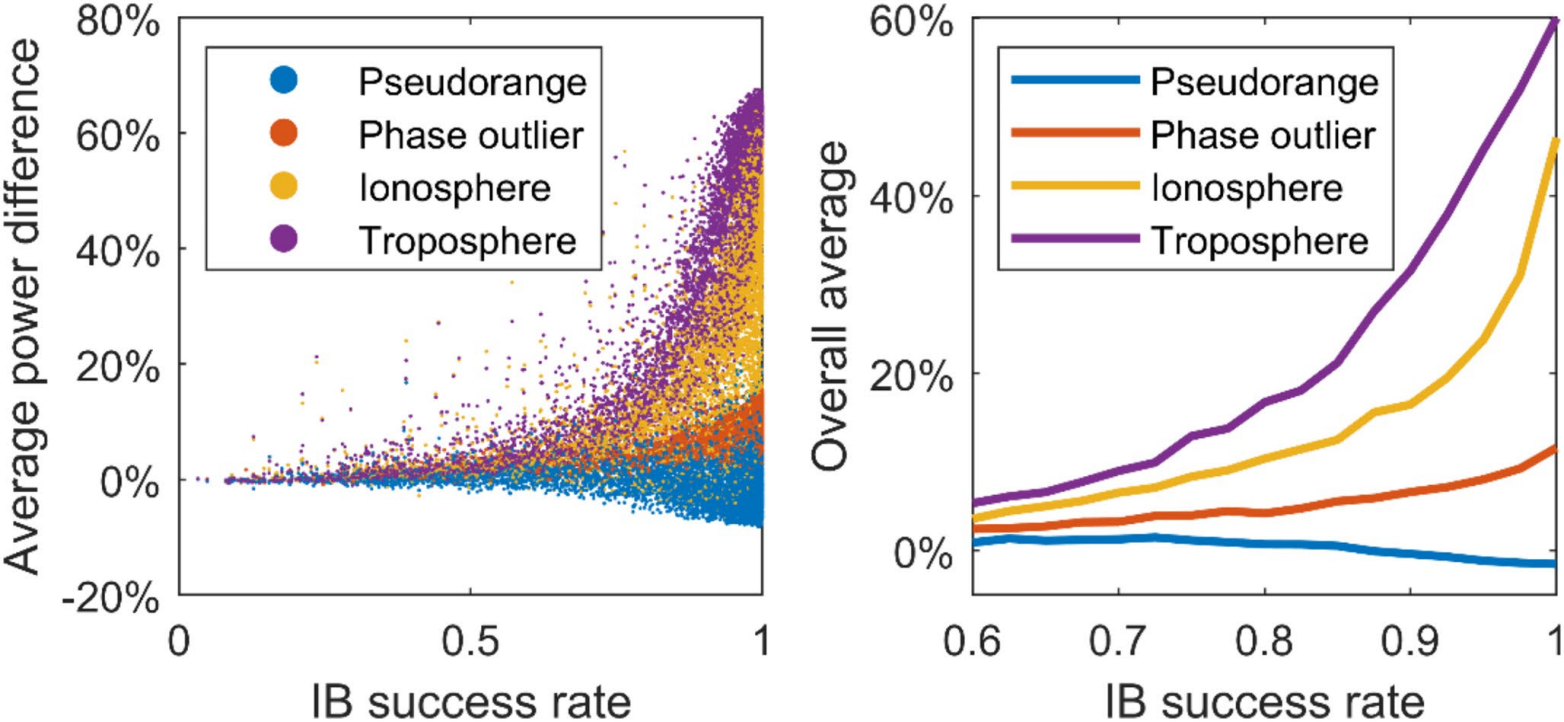
Left graph shows average power difference vs. IB success rate of corresponding experiment for four misspecifications, which are computed from power functions with $\:\alpha=0.01$. To obtain graph at right, we split success rate range $\:\left[0.6,\:1\right]$ into 16 intervals and compute overall average of power difference within each interval

Figure 11 (left) shows the average power differences obtained from the power functions with $\:\alpha\:=0.01$ for four misspecifications. Figure 11 (right) is obtained by splitting the success rate range $\:\left[0.6,\:1\right]$ into 16 intervals and compute the overall average of power difference within each interval, four of which are provided in Table 4. Compared with the AF detector, the AR detector performs better in detecting atmospheric delays and phase outliers. Improvement can be observed even though the ambiguity resolution success rate is not close to one. For the experiments with success rates in $\:[0.775,\:0.8]$, the detection powers are increased by 10% and 17% on average for ionosphere and troposphere delay detection. The improvement is significant when the success rate is close to one. For the experiments with success rates larger than 97.5%, the detection powers are increased by 12%, 47%, and 60% on average for phase outlier, ionosphere, and troposphere delay detection, respectively.

The results show that the improvement by the AR detector for the atmosphere delay is larger than that for the blunder in pseudorange, and the AR detector can perform poorer than the AF detector in the latter case. Teunissen (2024) explains the difference between the AF and AK detectors for detecting different types of misspecifications, which also helps to understand the performance of the AR detector since it lies between the AF and AK detectors. Although the AK test statistic has a larger noncentrality parameter, $\:{\lambda}_{\hat{e}\left(a\right)}>{\lambda}_{\hat{e}}$, it also has a larger critical value due to the stronger variability of $\:{\Vert\hat{\underline{e}}\left(a\right)\Vert}_{{Q}_{yy}}^{2}$. As a result, the AK detector can perform poorer than the AF detector if they have similar noncentrality parameters. For the ionosphere and troposphere delay detection, Teunissen (2024) shows that $\:{\lambda}_{\hat{e}\left(a\right)}$ is magnified by the factor $\:{\sigma}_{p}^{2}/{\sigma}_{\phi}^{2}$, which explains the superiority of the AR detector. For the blunder in pseudorange detection, according to Eq. (20) in Teunissen (2024), the difference between $\:{\lambda}_{\hat{e}\left(a\right)}$ and $\:{\lambda}_{\hat{e}}$ is driven by the projection of $\:{C}_{p}c$ in the range space of the $\:{B}_{p}$, where $\:{C}_{p}$ and $\:{B}_{p}$ refer to the submatrices of $\:C$ and $\:B$ containing rows for the pseudorange observables. With the principal angle (Björck and Golub 1973; Teunissen 1997) between the range space of $\:{C}_{p}$ and $\:{B}_{p}$, denoted as $\:{\theta}_{{C}_{p}B_p}$, the AK noncentrality parameter is magnified by a factor $\:\frac{1}{{sin}^{2}\left({\theta}_{{C}_{p}B_p}\right)}$ for one-dimensional $\:C$. The DD model in the previous dual-frequency experiment can be taken as an example where $\:{\theta}_{{C}_{p}B_p}$=$\:{55.2}^{^\circ}$ and $\:{\lambda}_{\hat{e}\left(a\right)}/{\lambda}_{\hat{e}}=1.48$. This factor is close to the ratio between the AK and AF critical values, which equals 1.79 for $\:\alpha=0.01$ in this example. For the blunder in pseudorange detection, the change in the noncentrality parameters $\:{\lambda}_{\hat{e}\left(a\right)}$ and $\:{\lambda}_{\hat{e}}$ is not significant compared with the change in critical values. Hence, the AK detector performs similarly to the AF detector for detecting the blunder in pseudorange, and so does the AR detector.

Overall, the AR detector is better as it performs similarly to the AF detector for the blunder in pseudorange detection and delivers higher detection powers for phase outliers and atmosphere delays.

### Multi-dimensional misspecifications

The performance of the AR detector for three multi-dimensional *(*$\:qD,\:q>1)$ misspecifications is evaluated and presented in this section. The simulation setup is the same as in the section ‘Performance for dual-frequency model’ where a dual-frequency (L1/L5) DD model is used. Detection for the following misspecifications is considered,

- carrier phase observables on L1 and L5 signals of satellite G05 are outliers (2D).
- blunders in pseudorange observables of two low-elevation satellites, G17 and G30 (4D).
- differential ionosphere delays affect the signals from the southwest direction (G05 and G24, 2D).

The power functions corresponding to the $\:qD$ misspecifications are $\:qD$ functions, which are evaluated on the $\:qD$ grid points of $$\:c=\left[\begin{array}{ccc}{c}_{1}&\:\cdots&\:{c}_{q}\end{array}\right]$$. The power functions plotted in this section are evaluated with $\:\alpha=0.01$ and $\:{N}_{1}={N}_{2}=2\times\:{10}^{5}$ for the Monte Carlo simulation of the critical values and power.

**Table 5:** Average power difference for $\:{q}{D}$ misspecification detection

Misspecification	$\:{\alpha}=0.005$	$\:{\alpha}=0.01$	$\:{\alpha}=0.05$
Phase outlier	31%	29%	22%
Blunder in pseudorange	7%	6%	5%
Ionosphere delay	68%	68%	56%

**Fig. 12:**
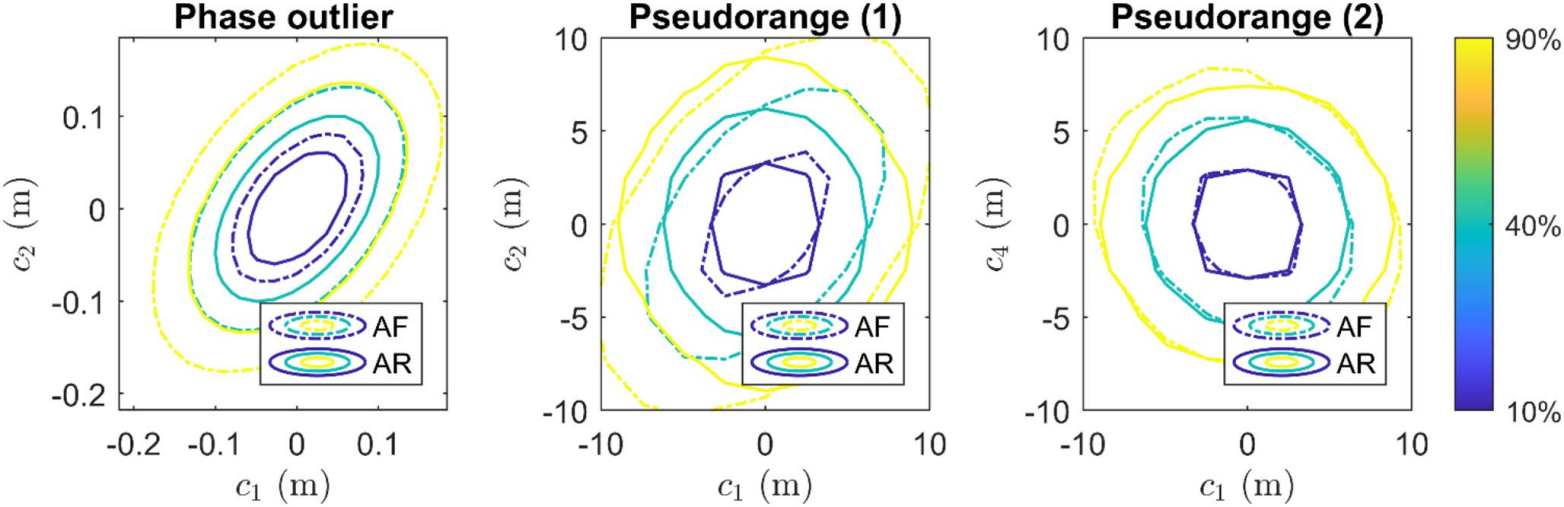
Left graph shows contour lines of AF and AR power functions for 2D phase outlier detection, evaluated on 400 grid points. $\:{c}_{1}$ and $\:{c}_{2}$ regarding to phase outliers of G05 on L1 and L5. Middle and right graphs show contour lines for marginal distributions of the 4D blunder in pseudorange power function, which is evaluated on $\:{13}^{4}=28,561$ grid points. $\:{c}_{1}$ and $\:{c}_{2}$ regarding to blunder in pseudoranges of G17 on L1 and L5. $\:{c}_{4}$ is size of blunder in pseudorange of G30 on L5

**Fig. 13:**
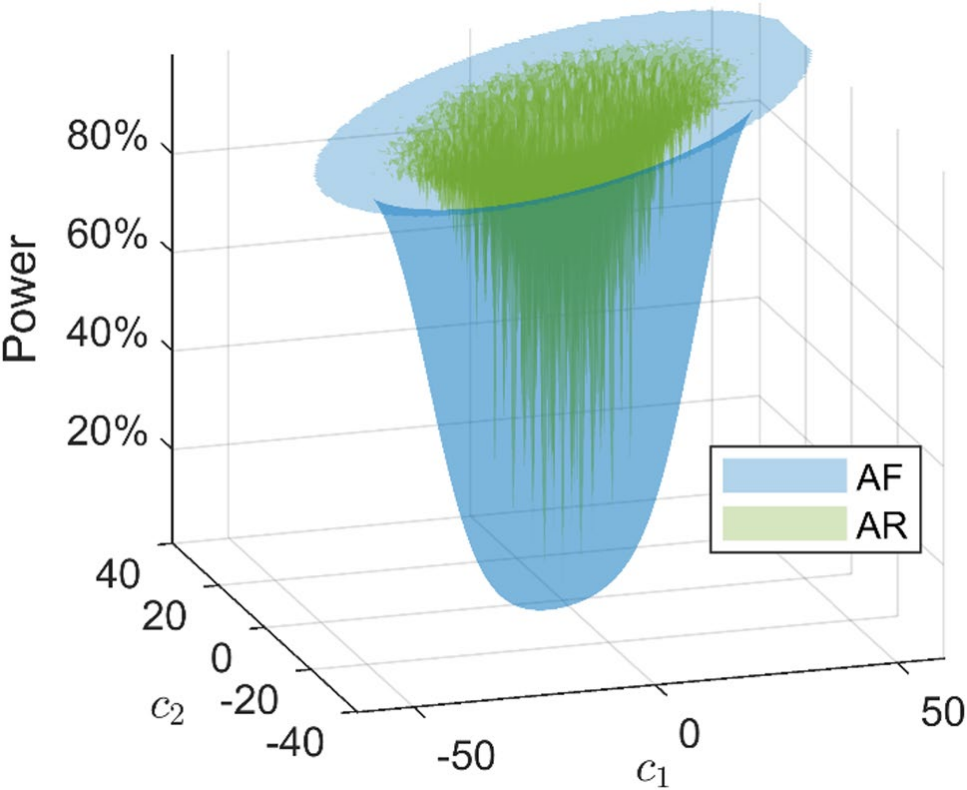
AF and AR power functions for 2D ionosphere delay detection. Only powers smaller than 0.999 are shown for clarity of the figure. $\:{c}_{1}$ and $\:{c}_{2}$ are size (in TECU) of the differential ionosphere delays corresponding to G05 and G24

Figure 12 (left) shows the contour lines of the AF and AR detector for 2D phase outliers. The AR contour lines are more concentrated, indicating that the AR detector provides a higher detection power for phase outliers of the same size. Table 5 presents that the average power difference between the AR and AF detectors is 29% for $\:\alpha=0.01$ in this experiment. The middle and right graphs in Fig. 12 show contour lines for two marginal distributions of the 4D blunder in pseudorange power function. Similar to the results in the one-dimensional case, the AR and AF detectors perform similarly. The average increase in the detection power is 6% for $\:\alpha=0.01,$ as is shown in Table 5. Figure 13 shows the power functions of the 2D differential ionosphere delay detection. We observe an oscillation of the AR power function, which is higher than that of the AF detector. Table 5 shows that the average improvement in the detection power is 68% for $\:\alpha=0.01$. The AR detector provides higher detection powers than the AF detector, especially for ionosphere delay detection in multi-dimensional misspecification experiments.

### Summary and conclusions

In this contribution, we evaluated the performance of the ambiguity-resolved (AR) detector for the GNSS relative positioning model by obtaining the detection powers as functions of the misspecifications’ size. We compared its performance with the ambiguity-float (AF) and ambiguity-known (AK) detectors.

We first presented the differential observation model under the null ($\:{H}_{0}$) and alternative ($\:{H}_{a}$) hypotheses. The model under $\:{H}_{a}$ contains an additional term to account for misspecifications and is used to evaluate the detection power for the misspecification. The performance of detecting the blunders in pseudorange, phase outliers, ionosphere and troposphere delays, and constant bias was evaluated.

Then, we described how the detectors could be implemented and how to obtain power functions. The distribution of the AR test statistic cannot be evaluated analytically. We presented the procedure to obtain the AR critical value and detection power using Monte Carlo simulation. Moreover, we introduced how to assess the uncertainty of the simulated critical value and detection power.

We conducted simulation experiments to evaluate the performance of the AR detector. We started with three experiments of detecting one-dimensional misspecifications. We first conducted a single-frequency GPS experiment with an 80.5% double-differenced (DD) ambiguity resolution success rate, in which the distributions of the test statistics under $\:{H}_{0}$ and $\:{H}_{a}$ were presented. It is shown by the power functions that the AR detector has larger detection power than the AF detector, even though the success rate is not close to one. Then, we conduct a dual-frequency GPS experiment with a 97.4% DD success rate. The difference between the AR and AF detection power of atmosphere delays is over 49% on average. In these two experiments, we also find that the AR power function differs from the AK power function. Hence, one should not predict the performance of the AR detector relying on the assumed probabilistic property of the AK detector as it is too optimistic.

Following that, we obtained power functions on 25 user locations, with five different observation models and 72 satellite geometries per location per model. For the experiments with success rates larger than 97.5%, the AR detector performs similarly to the AF detector for the blunder in pseudorange detection, increases the detection power by 12% on average for the phase outlier detection, and increases the detection power remarkably by 46% and 60% on average for the ionosphere and troposphere detection, respectively. Finally, we presented experiments of detecting three multi-dimensional misspecifications. The AR detector performs better than the AF detector in all the experiments, especially for the two-dimensional ionosphere delay detection, where the powers are increased by 68% on average for $\:\alpha=0.01$.

The simulation experiments show that the AR detector can provide a higher detection power than the AF detector even if the ambiguity resolution success rate is not close to one. For models with low success rates, although the ambiguity resolution cannot be used by the parameter estimation, the AR detector can contribute to model validation.

## Data Availability

The GNSS orbit product used in this research can be downloaded from https://cddis.nasa.gov/archive/gnss/products/.
